# The Influence of Expectancy Level and Personal Characteristics on Placebo Effects: Psychological Underpinnings

**DOI:** 10.3389/fpsyt.2019.00020

**Published:** 2019-02-05

**Authors:** Lili Zhou, Hua Wei, Huijuan Zhang, Xiaoyun Li, Cunju Bo, Li Wan, Xuejing Lu, Li Hu

**Affiliations:** ^1^CAS Key Laboratory of Mental Health, Institute of Psychology, Chinese Academy of Sciences, Beijing, China; ^2^Department of Psychology, University of Chinese Academy of Sciences, Beijing, China; ^3^Key Laboratory of Cognition and Personality, Ministry of Education, Faculty of Psychology, Southwest University, Chongqing, China; ^4^Department of Pain Management, The State Key Clinical Specialty in Pain Medicine, The Second Affiliated Hospital of Guangzhou Medical University, Guangzhou, China

**Keywords:** expectancy, placebo analgesia, gender, dispositional optimism, anxiety state

## Abstract

Placebo effects benefit a wide range of clinical practice, which can be profoundly influenced by expectancy level and personal characteristics. However, research on the issue of whether these factors independently or interdependently affect the placebo effects is still in its infancy. Here, we adopted a 3-day between-subject placebo analgesia paradigm (2-day conditioning and 1-day test) to investigate the influence of expectancy levels (i.e., No, Low, and High) and personal characteristics (i.e., gender, dispositional optimism, and anxiety state) on placebo effects in 120 healthy participants (60 females). Our results showed that the reduction of pain intensity in the test phase was influenced by the interaction between expectancy and gender, as mainly reflected by greater reductions of pain intensity in females at Low expectancy level than females at No/High expectancy levels, and greater reductions of pain intensity in males than in females at High expectancy level. Additionally, the reduction of pain unpleasantness was not only modulated by the interaction between expectancy and gender, but also by the interaction between expectancy and dispositional optimism, as well as the interaction between expectancy and anxiety state. Specifically, participants who were more optimistic in Low expectancy group, or those who were less anxious in High expectancy group showed greater reductions of pain unpleasantness. To sum up, we emphasized on regulating the expectancy level individually based on the assessment of personal characteristics to maximize placebo effects in clinical conditions.

## Introduction

Placebo commonly refers to an inert substance or a medicinally inactive treatment that can generate clinically-useful effects. A person who receives a placebo treatment usually experiences actual improvements in his/her physical condition, which is well-known as the *placebo effect*. The placebo effect can be beneficial in a wide range of clinical situations, such as modulating the therapeutic effects of deep brain stimulation on Parkinson's disease, generating antidepressant responses in depression, and reducing unpleasantness in patients with anxiety (1, 2). It can also enhance the effectiveness of physical interventions (3–5) and provide an alternative approach to avoid side effects of drug treatments (6, 7).

Although placebo effect is a complex phenomenon that can be affected by multiple factors (e.g., memory, motivation, anxiety, learning, patient-provider interaction, and previous treatment experience) (1, 8–10), response expectancy has been recognized as one of the main psychological mechanisms underlying this effect (11). Response expectancy has been defined as the expectancy to the occurrence of non-volitional responses (i.e., responses experienced as occurring automatically without volitional efforts, including fear, sadness, sexual arousal, pain, etc.) to situational cues (12). According to *Response Expectancy Theory*, such response expectancy could affect the probability that an individual would engage in a particular behavior (e.g., increased/decreased pain responses), as non-volitional responses have positive and negative reinforcement values (12). Consistent with this theory, accumulating evidence has shown that the placebo effect (e.g., placebo analgesia) could be altered by changing individual expectancy (3, 7, 13–19).

In general, response expectancy is composed of two distinct aspects: (1) the expected magnitude of a change (i.e., expectancy level), and (2) the subjective probability that the change will occur (i.e., individual belief) (12). With regards to the first aspect of response expectancy, a greater placebo effect is usually associated with a higher level of positive expectancy (4, 5, 20–23). However, these observations are not guaranteed under some circumstances. For example, in laboratory settings, even individual expectation of placebo effects has been successfully acquired during the classical conditioning phase, unrealistically high expectancy that does not match with one's present experience would weaken individual belief in the placebo treatment during the test phase (24).

In terms of the second aspect of response expectancy, previous studies have demonstrated that individual belief in the current experience has a critical influence on response expectancy through learning mechanisms (21, 24). Such a belief is easily affected by personal characteristics, thus contributing to the differentiation of individual expectancy (25, 26), and subsequently leading to individual variability in response to placebos (27, 28). For instance, gender has been verified as a factor contributing to the variability of placebo effects—some studies suggested that males reported greater pain reductions after placebo treatments compared to females (29–31), whereas other studies described a better respondence to placebos in females than in males (29, 32–34). Additionally, the influence of other personal characteristics on placebo responses has been frequently reported in the literature. For example, dispositional optimism, referred to a generalized positive outcome expectancy for the future (35), is inextricably linked to proneness of increased placebo effects (36, 37). Comparatively, individuals with low anxiety level are more likely to respond to a placebo treatment (36).

However, research on the issue of whether expectancy level and personal characteristics independently or interdependently affect the placebo effects is still in its infancy. Here, we adopted a between-subject placebo analgesia paradigm to test the influence of expectancy levels (i.e., No, Low, and High) and personal characteristics (i.e., gender, dispositional optimism, and anxiety state) on placebo effects.

## Materials and Methods

### Participants

A total of 120 healthy, right-handed participants (60 females) were recruited from the local community. None of them reported a history of illness or concurrent medication. Participants were informed that they were attending a study aimed to test the effect of lidocaine (a local anesthetic that could be topically applied on the skin) on alleviating pain, and they were asked not to consume products containing caffeine, alcohol, or nicotine at least 12 h before the experiment. All the participants gave their written informed consents and were told their rights to discontinue participation at any time during the study. Each participant was randomly assigned to one of the three experimental groups divided by the manipulated expectancy levels (i.e., No, Low, and High) during the Conditioning phase (as described below), with 40 participants (20 females) in each group. After the whole experiment, all participants were fully debriefed.

### Experimental Materials

#### Pain Stimuli

The electrical pain stimuli were delivered using a constant-current stimulator (model DS7A; Digitimer, UK) with three stainless steel concentric bipolar needle electrodes (38, 39). Pain stimuli were intraepidermal electrical pulses delivered to the inner side of the left forearm through the electrodes (located according to an equilateral triangle shape), which have been proved to preferentially activate the Aδ nociceptive fibers in the superficial skin layers (40, 41). Each electrode consisted of a needle cathode (length = 0.1 mm, diameter = 0.2 mm) surrounded by a cylindrical anode (diameter = 1.4 mm). Each stimulus consisted of 100 rapidly succeeding constant-current, square-wave pulses at 50 Hz (0.5-ms duration for each pulse).

#### Dispositional Optimism

The Chinese version of the Life Orientation Test-Revised (LOT-R) was adopted to assess participants' dispositional optimism, as its reliability has been well-established (Cronbach alpha of positive subscale = 0.73, *N* = 479; Cronbach alpha of negative subscale = 0.82, *N* = 479) (42). In the current sample, the reliability of the scale was satisfactory (Cronbach alpha = 0.66, *N* = 120).

#### Anxiety State

The state subscale of Chinese version of State-Trait Anxiety Inventory (STAI-S) was adopted to assess participants' anxiety state. The reliability of the Chinese version of STAI-S (Cronbach alpha = 0.90, *N* = 2,150) (43) has been well-established. Notably, the reliability of the subscale in the current sample was satisfactory (STAI-S: Cronbach alpha = 0.89, *N* = 120).

### Experimental Procedure

A randomized, single-blinded between-subject experimental paradigm of placebo analgesia was adopted in the present study (7). Participants were firstly familiarized with the electrical stimulation prior to the formal experiment. The stimulus intensities were adjusted individually using the method of limits, to identify the thresholds for each participant that would elicit a low sensation (~2 rating), moderate sensation (~4 rating), and high sensation (~6 rating) on an 11-point self-report Numeric Rating Scale (NRS, 0 = no sensation, 10 = unbearable pain). Specifically, the stimuli at ~2 rating elicited a non-painful sensation, whereas the stimuli at ~4 and ~6 ratings elicited a painful pinprick sensation. Once these stimulus intensities were determined, a randomized sequence of pain stimuli with different intensities was delivered to participants until they were able to reliably distinguish the intensities of these stimuli. Notably, these determined stimuli with varied intensities were used during the conditioning procedure (see Conditioning Phase section) to ensure a successful manipulation of expectancy level during the experiment.

**Figure 1:**
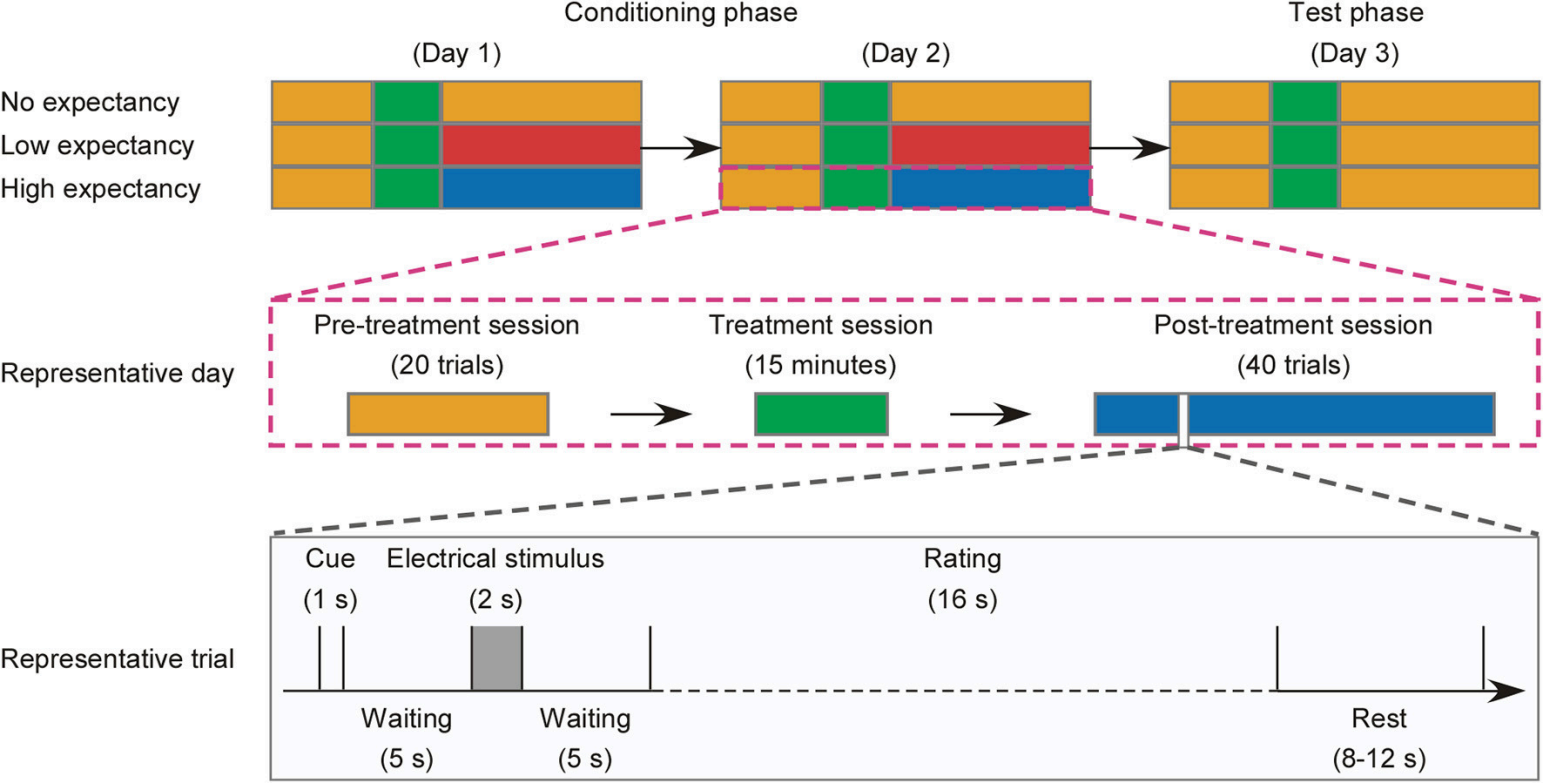
Flowchart of the experimental design. **(Top)** For each experimental group (No, Low, and High expectancy), the experiment consisted of two phases: Conditioning phase (Day 1 and Day 2) and Test phase (Day 3), and each day consisted of three sessions: a pre-treatment session, a treatment session, and a post-treatment session. Sessions, in which the electrical stimuli elicit a low sensation at ~2 rating, moderate sensation at ~4 rating, and high sensation at ~6 rating on the 11-point Numerical Rating Scale, are marked in blue, red, and orange, respectively. Treatment sessions are marked in green. **(Middle)** The experimental procedure in a representative day contained a 20-trial pre-treatment session, a 15 min treatment session, and a 40-trial post-treatment session, which are marked in orange, green, and blue, respectively. **(Bottom)** A representative trial of the pre-treatment session or the post-treatment session was starting with a 1-s cue, followed by a 5 s waiting, a 2 s electrical stimulus, another 5 s waiting, and a 16 s rating of the perception of pain intensity, unpleasantness, anxiety level, and satisfaction of drug efficacy (post-treatment session in Low and High expectancy groups). The trial was ended with a rest of 8–12 s.

The experiment consisted of two phases in three consecutive days: *Conditioning phase* (Day 1 and Day 2) and *Test phase* (Day 3). On each day, participants underwent three sessions: (1) a pre-treatment session, (2) a treatment session, and (3) a post-treatment session (see Figure 1). To rule out possible confounding effects related to the gender of experimenter (44, 45), half of participants in each group with an equal number of males (*n* = 20) and females (*n* = 20) were instructed by a female experimenter, while the rest were guided by a male experimenter. Both female and male experimenter wore white coats and had received systematic training of procedure prior to the formal experiment.

#### Conditioning Phase

The Conditioning phase started with a pre-treatment session consisting of 20 trials. Each trial started with a 1 s white fixation centered on the screen with a black background. After a 5 s waiting, an electrical stimulus at ~6 ratings (0.80 ± 0.29 mA) lasting for 2 s was delivered to the left forearm of the participant. Being waiting for another 5 s, participants were required to verbally rate the perceived intensity (0 = no sensation, 10 = unbearable pain) and unpleasantness (0 = not unpleasant, 10 = extremely unpleasant) of pain evoked by the electrical stimulus (with 8 s per each rating, 16 s in total). The inter-trial interval varied between 8 and 12 s. The stimulus intensity in the pre-treatment session was identical across groups and the whole session lasted for ~16 min.

In the treatment session, a non-active skin cream was applied on the palmar side of the participant's left forearm. Being waiting for 5 min, participants were instructed to remove the cream and have a 10 min rest. Meanwhile, in order to strength expectancy level, participants were given one of the following verbal interventions, depending on treatment assignment:

(1) participants in No expectancy group were told that “*the skin cream is ineffective to relieve or eliminate pain*”;
(2) participants in Low expectancy group were told that “*the skin cream can reduce but not eliminate pain*”;
(3) participants in High expectancy group were told that “*the skin cream can completely eliminate pain*.”

The treatment session lasted for ~15 min.

The Conditioning phase ended with a post-treatment session consisting of 40 trials. The procedure was identical to the pre-treatment session, except that different intensities of electrical stimuli were set for different groups: inducing a painful sensation at ~6 rating (0.69 ± 0.16 mA) for No expectancy group, at ~4 rating (0.47 ± 0.17 mA) for Low expectancy group, and a non-painful sensation at ~2 rating (0.28 ± 0.08 mA) for High expectancy group. Such changes of stimulus intensity for different groups were intended to strengthen the power of verbal intervention to response expectancy, which has been frequently applied in previous placebo-related studies (46–48). The post-treatment session lasted for ~37 min.

#### Test Phase

The Test phase also consisted of a pre-treatment session, a treatment session, and a post-treatment session. The procedure of this phase was identical to the *Conditioning phase*, except that the intensity of electrical stimuli applied in the post-treatment session was identical for all participants across groups, i.e., inducing a painful pinprick sensation at ~6 rating (0.78 ± 0.26 mA). Participants were first required to complete the psychological questionnaires upon arriving to the laboratory on the Test day. To make sure that the expectancy manipulation was successful, participants in the Low and High Expectancy groups were required to verbally rate the strength of expectancy to drug efficacy on an 11-point NRS (0 = without any expectancy, 10 = full expectancy) at the end of the test. The average ratings of expectancy to drug efficacy were significantly different between groups (Low: 6.90 ± 1.31, High: 8.39 ± 0.80, *t* = −6.11, *P* < 0.001), indicating a successful expectancy manipulation. For participants in No expectancy group, the expectancy to drug efficacy was not assessed to avoid extra response bias to the expectancy manipulation.

### Statistical Analysis

To assess the magnitude of placebo effects, we calculated the changes of subjective pain intensity and unpleasantness by subtracting the ratings in the post-treatment session from those in the pre-treatment session in Test phase (49). To demonstrate the influence of personal characteristics on the modulation of expectancy level on placebo effects, we performed a statistical analysis using a “split into three subgroups” strategy (13-14-13 split). Specifically, we sorted the LOT-R scores in ascending order and split the data into low (13 participants), middle (14 participants), and high LOT-R subgroups (13 participants) for each experimental condition. Following, we performed three-way analyses of variance (ANOVAs) on the indicators of placebo effects, with “expectancy level” (No, Low, and High), “dispositional optimism (LOT-R)” (low and high), and “gender” (female and male) as between-subject factors. Likewise, scores of anxiety state (STAI-S) were sorted and analyzed using the same statistical strategy. The statistical *P*-values were adjusted with Greenhouse-Geisser correction to avoid violation of the sphericity assumption, when necessary. *Post hoc* pairwise comparisons were performed with Bonferroni adjustments, when the main effects or interactions reach statistical significance. The effect size and statistical power in the present sample were estimated by partial eta-squared and 1-β, respectively. For partial eta-squared ($\eta_{p}^{2}$), an effect size of 0.0099 is deemed as a “small” effect, around 0.0588 as a “medium” effect, and 0.1379 to infinity as a “large” effect (50). For 1- β, 0.8 is the commonly acceptable statistical power. To detect the effectiveness of sample size used in the current study, we performed a prior computation on the required sample size using G^*^power (an online free software for power analysis, available at http://www.gpower.hhu.de/en.html) by setting statistical power at 0.8 with large effect size ($\eta_{p}^{2}$ = 0.1379). The result showed a minimal sample size of 64 in total to detect the main effects and interactions between independent variables, which indicated that our sample size (*N* = 120) was enough to detect these effects. All statistical analyses were carried out in SPSS 22.0 statistical analysis package (SPSS Inc., New York, USA). Statistical threshold was set at 0.05.

## Results

### Participant Characteristics

Participant characteristics for each experimental group are summarized in Table 1. The age was not significantly associated with “expectancy level” (No, Low, and High) and “gender” (female and male) [*F*_(2, 114)_ = 2.84, *P* = 0.06, $\eta_{p}^{2}$ = 0.05]. This result, together with the counterbalanced experimental design for gender, indicated that all the participants across groups were age- and gender-matched, thus avoiding possible bias when assessing placebo effects.

**Table 1:** Characteristics of participants in each experimental group (data are expressed as mean ± standard deviation, M ± SD).

**Group**	**Gender**	***N***	**Age**
No expectancy	F	20	20.20 ± 1.15
	M	20	21.30 ± 1.38
Low expectancy	F	20	20.95 ± 1.28
	M	20	21.55 ± 1.73
High expectancy	F	20	21.40 ± 1.39
	M	20	21.00 ± 1.59

*F, Female; M, Male*.

### Influence of Expectancy Level, Dispositional Optimism, and Gender on Placebo Effects

**Figure 2:**
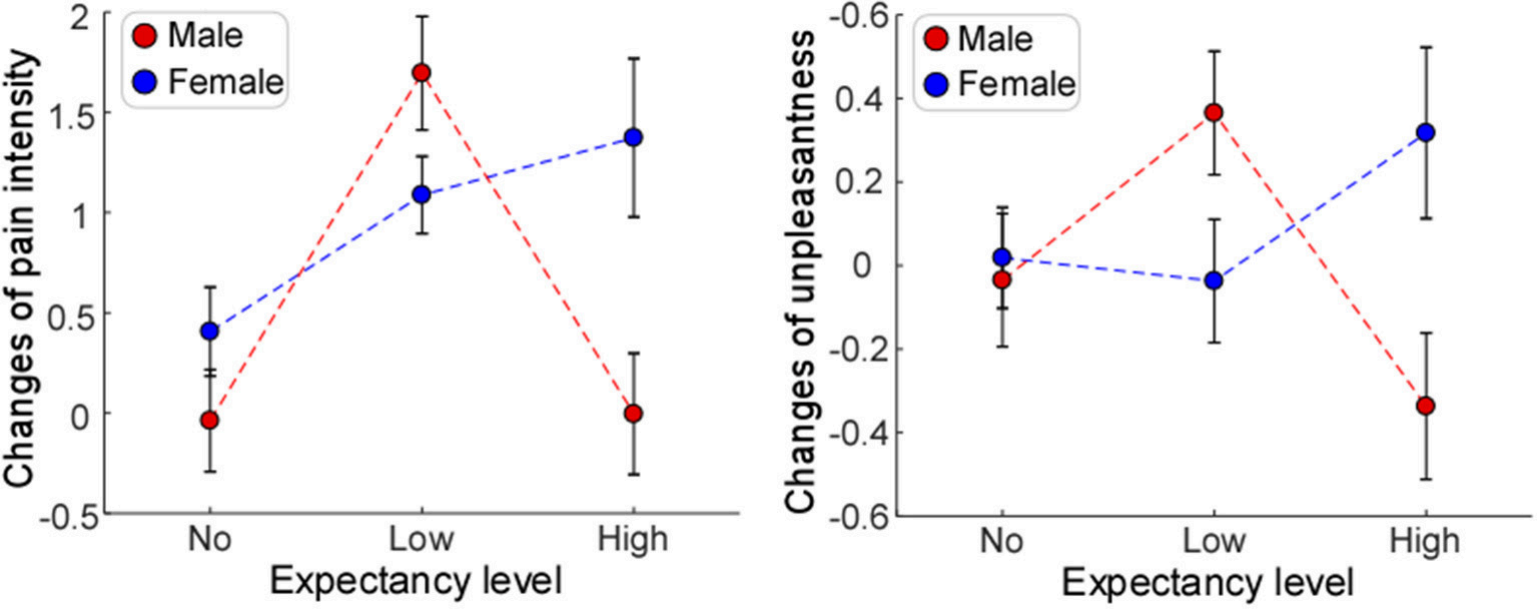
The influences of expectancy level and gender on placebo effects. Significant main effect of expectancy level was only observed for changes of pain intensity **(Left)**. Significant interactions of expectancy level and gender were observed for changes of pain intensity **(Left)** and unpleasantness **(Right)**. Error bars indicate standard error, and data from female and male participants are marked in blue and red, respectively.

**Figure 3:**
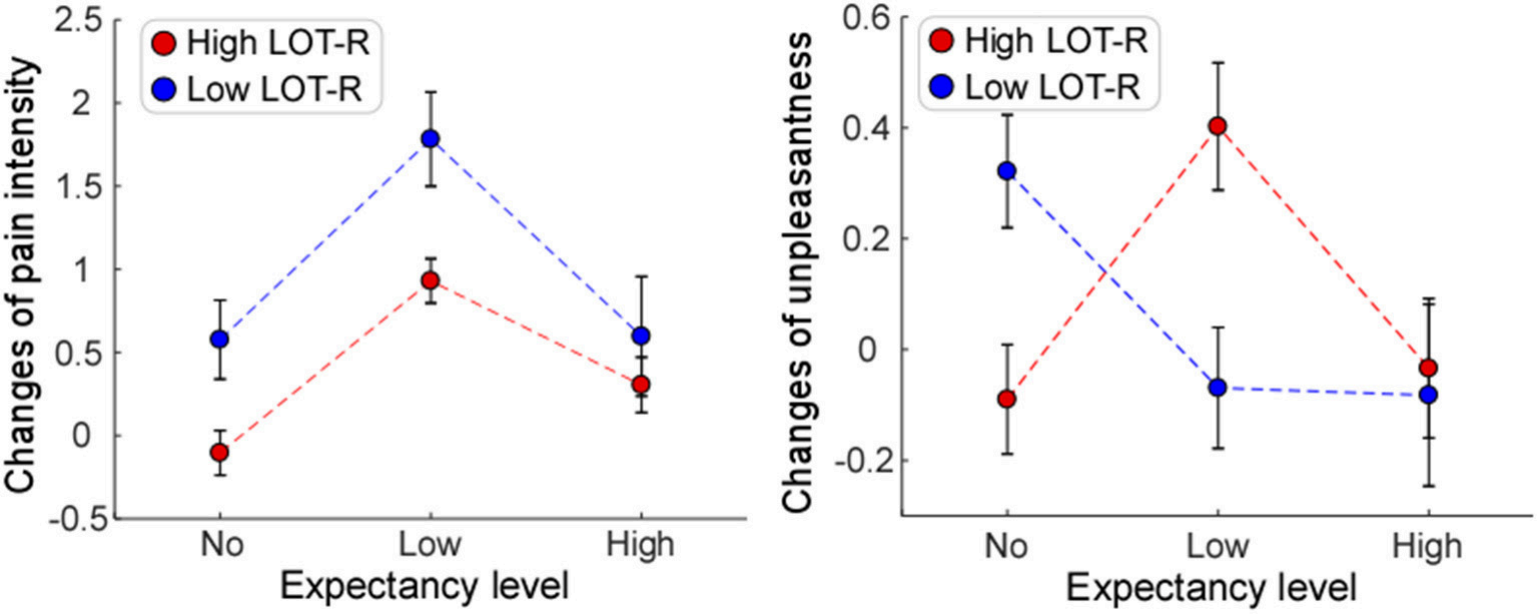
The influences of expectancy level and dispositional optimism (LOT-R) on placebo effects. Significant main effect of expectancy level was only observed for changes of pain intensity **(Left)**. Significant interaction of expectancy level and dispositional optimism (LOT-R) was observed for changes of unpleasantness **(Right)**, but not for changes of pain intensity **(Left)**. Error bars indicate standard error, and data from participants with low and high scores of Life Orientation Test-Revised (Low LOT-R and High LOT-R) are marked in blue and red, respectively.

Significant main effects of “expectancy level” [*F*_(2, 72)_ = 7.06, *P* = 0.002, η_*p*_^2^ = 0.172] and “dispositional optimism (LOT-R)” [*F*_(1, 72)_ = 5.18, *P* = 0.026, η_*p*_^2^ = 0.071] were observed, while no significant main effect of “gender” [*F*_(1, 114)_ = 0.04, *P* = 0.848, η_*p*_^2^ = 0.001] was showed in the reduction of pain intensity (see Table 2). *Post hoc* comparisons on “expectancy level” showed that participants in Low expectancy group elicited a greater reduction of pain intensity than both No (*P* < 0.001) and High (*P* = 0.055) expectancy groups, while the latter two had no significant difference (*P* = 0.29). Neither the interaction between “expectancy level” and “dispositional optimism (LOT-R)” [*F*_(1, 72)_ = 0.79, *P* = 0.460, η_*p*_^2^ = 0.023] (Figure 3, left panel), nor the interaction between “dispositional optimism (LOT-R)” and “gender” [*F*_(2, 72)_ = 0.20, *P* = 0.655, η_*p*_^2^ = 0.003] (see Figure 2, left panel) was significant. However, the interaction between “expectancy level” and “gender” [*F*_(2, 114)_ = 4.29, *P* = 0.018, η_*p*_^2^ = 0.112] was significant. *Post-hoc* comparison on this interaction revealed that (1) female participants in the Low expectancy group reported a greater reduction of pain intensity due to placebo treatment than females in No (*P* < 0.001) and High (*P* = 0.001) expectancy groups; (2) for participants in High expectancy group, males reported a greater reduction of pain intensity due to placebo treatment than females (*P* = 0.01).

**Table 2:** The changes of pain intensity from pre-treatment to post-treatment sessions in all experimental groups (data are expressed as M ± SD).

**Personal characteristics**		**Response expectancy**		
		**No**	**Low**	**High**
Gender	F	−0.04 ± 1.14	1.70 ± 1.27	−0.004 ± 1.35
	M	0.41 ± 0.99	1.09 ± 0.87	1.37 ± 1.77
LOT-R	Low	0.58 ± 1.21	1.78 ± 1.44	0.59 ± 1.83
	High	−0.10 ± 0.68	0.93 ± 0.68	0.31 ± 0.85
STAI-S	Low	−0.22 ± 1.16	0.94 ± 0.69	1.21 ± 1.83
	High	0.39 ± 1.31	1.54 ± 1.38	0.75 ± 1.89

*F, Female; M, Male; LOT-R, Life Orientation Test-Revised; STAI-S, State subscale of State-Trait Anxiety Inventory. Changes of pain intensity were obtained by subtracting the ratings in post-treatment sessions from those in pre-treatment sessions*.

In contrast, main effects of “expectancy level” [*F*_(2, 72)_ = 1.00, *P* = 0.374, η_*p*_^2^ = 0.029], “dispositional optimism (LOT-R)” [*F*_(1, 72)_ = 0.05, *P* = 0.832, η_*p*_^2^ = 0.001], and “gender” [*F*_(1, 72)_ = 0.03, *P* = 0.874, η_*p*_^2^ < 0.001] were not significant on the reduction of pain unpleasantness (see Table 3). Except of non-significant interaction between “dispositional optimism (LOT-R)” and “gender” [*F*_(2, 72)_ = 0.78, *P* = 0.379, η_*p*_^2^ = 0.011], both the interaction between “expectancy level” and “dispositional optimism (LOT-R)” [*F*_(2, 72)_ = 3.26, *P* = 0.044, η_*p*_^2^ = 0.084] (Figure 3, right panel), and the interaction between “expectancy level” and “gender” [*F*_(2, 72)_ = 3.38, *P* = 0.040, η_*p*_^2^ = 0.091] (Figure 2, right panel) were statistically significant. *Post-hoc* comparison on the interaction between “expectancy level” and “dispositional optimism (LOT-R)” showed that (1) for participants with high LOT-R scores, those in Low expectancy group experienced a greater reduction of unpleasantness due to placebo treatment than those in No expectancy group (*P* = 0.046); (2) for Low expectancy group, participants with high LOT-R scores had a tendency to report a greater reduction of unpleasantness than those with low LOT-R scores (*P* = 0.056; see Table 3). Similar to the results of pain intensity, *post-hoc* pairwise comparisons showed that (1) female participants in the Low expectancy group reported a greater reduction of unpleasantness due to placebo treatment than those in High expectancy group (*P* = 0.039); (2) for participants in High expectancy group, males tended to report a greater reduction of unpleasantness due to placebo treatment than females (*P* = 0.073).

**Table 3:** The changes of pain unpleasantness from pre-treatment to post-treatment sessions in all experimental groups (data are expressed as M ± SD).

**Personal characteristics**		**Response expectancy**		
		**No**	**Low**	**High**
Gender	F	−0.03 ± 0.71	0.36 ± 0.66	−0.34 ± 0.78
	M	0.02 ± 0.54	−0.04 ± 0.66	0.32 ± 0.92
LOT-R	Low	0.32 ± 0.52	−0.07 ± 0.56	−0.08 ± 0.84
	High	−0.09 ± 0.50	0.40 ± 0.58	−0.03 ± 0.64
STAI-S	Low	−0.25 ± 0.56	0.07 ± 0.72	0.47 ± 1.11
	High	0.35 ± 0.71	0.21 ± 0.81	−0.13 ± 0.86

*F, Female; M, Male; LOT-R, Life Orientation Test-Revised; STAI-S, State subscale of State-Trait Anxiety Inventory. Changes of pain unpleasantness were obtained by subtracting the ratings in post-treatment sessions from those in pre-treatment sessions*.

### Influence of Expectancy Level, Anxiety State, and Gender on Placebo Effects

**Figure 4:**
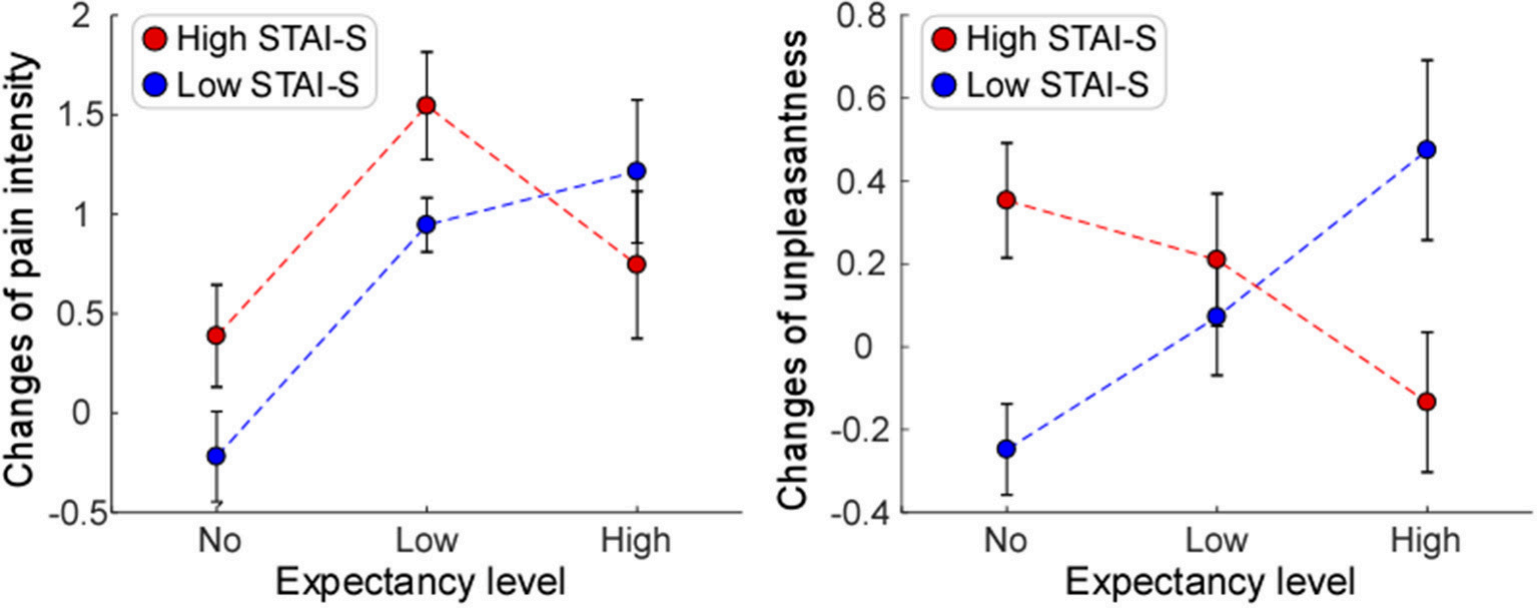
The influences of expectancy level and anxiety state (STAI-S) on placebo effects. Significant main effect of expectancy level was only observed for changes of pain intensity **(Left)**. Significant interaction of expectancy level and anxiety state (STAI-S) was observed for changes of unpleasantness **(Right)**, but not for changes of pain intensity **(Left)**. Error bars indicate standard error, and data from participants with low and high scores of State subscale of State-Trait Anxiety Inventory (Low STAI-S and High STAI-S) are marked in blue and red, respectively.

For the reduction of pain intensity, there was a significant main effect of “expectancy level” [*F*_(2, 72)_ = 4.14, *P* = 0.02, η_*p*_^2^ = 0.026]. *Post-hoc* comparisons showed that participants in Low expectancy group reported a greater reduction of pain intensity than those in No expectancy group (*P* = 0.007). No significant main effect of “gender” [*F*_(1, 72)_ = 3.07, *P* = 0.082, η_*p*_^2^ = 0.09] or “anxiety state (STAI-S)” [*F*_(1, 72)_ = 1.21, *P* = 0.26, η_*p*_^2^ = 0.02] was found. No significant interaction between “expectancy level” and “anxiety state (STAI-S)” [*F*_(2, 72)_ = 1.68, *P* = 0.19, η_*p*_^2^ = 0.05] (see Figure 4, left panel), or between “anxiety state” and “gender” [*F*_(2, 72)_ = 1.27, *P* = 0.26, η_*p*_^2^ = 0.02] was observed. However, the interaction between “expectancy level” and “gender” was significant [*F*_(2, 72)_ = 4.04, *P* = 0.02, η_*p*_^2^ = 0.11]. *Post-hoc* comparison on this interaction revealed the same pattern as the results reported in the previous section (“Influence of expectancy level, dispositional optimism, and gender on placebo effects”).

With regard to the reduction of unpleasantness, no main effect of “expectancy level” [*F*_(2, 72)_ = 0.12, *P* = 0.891, η_*p*_^2^ = 0.003], “anxiety state (STAI-S)” [*F*_(1, 72)_ = 0.07, *P* = 0.796, η_*p*_^2^ = 0.001], or “gender” [*F*_(1, 72)_ = 1.28, *P* = 0.263, η_*p*_^2^ = 0.018] was observed. The interaction between “expectancy level” and “anxiety state (STAI-S)” [*F*_(2, 72)_ = 4.05, *P* = 0.022, η_*p*_^2^ = 0.106] (Figure 4, right panel), and the interaction between “expectancy level” and “gender” [*F*_(2, 72)_ = 4.49, *P* = 0.015, η_*p*_^2^ = 0.117] (Figure 2, right panel) were significant. *Post-hoc* pairwise comparisons on the interaction between “expectancy level” and “anxiety state (STAI-S)” showed that for participants with low STAI-S scores, those in High expectancy group felt less pain unpleasantness due to the placebo treatment than those in No expectancy group (*P* = 0.027). *Post-hoc* comparison on interaction between “expectancy level” and “gender” revealed the same pattern as the results reported in the previous section (“Influence of expectancy level, dispositional optimism, and gender on placebo effects”). No significant interaction was observed between “anxiety state (STAI-S)” and “gender” [*F*_(2, 72)_ = 2.39, *P* = 0.127, η_*p*_^2^ = 0.034].

## Discussion

In the present study, we demonstrated that placebo effects were not only influenced by expectancy level or personal characteristics alone, but also depended on their interactions. Specifically, we observed that the reductions of pain intensity and pain unpleasantness in the Test phase were influenced by the interaction between expectancy level and gender, as mainly reflected by greater reductions of pain intensity and pain unpleasantness in females at Low expectancy level than females at No/High expectancy levels, and greater reductions of pain intensity and pain unpleasantness in males than in females at High expectancy level. Additionally, the reduction of pain unpleasantness was modulated by the interaction between expectancy level and dispositional optimism, as well as the interaction between expectancy level and anxiety state. Participants who were more optimistic in Low expectancy group, or those who were less anxious in High expectancy group showed greater reductions of pain unpleasantness.

Firstly, placebo effects, defined as the reduction of pain intensity or unpleasantness, depended on the interaction between expectancy level and gender. We found female participants elicited maximal placebo effects in Low rather than No and High expectancy groups, which is in line with the previous study showing that placebo effects, as quantified by systolic blood pressure, alertness, and tension, were stronger at the moderate expectancy level than at the extremely low and high expectancy levels (24). In other words, a realistically reasonable expectancy, rather than the unrealistic expectancy level, is more likely to enhance individual belief of the treatment, which is essential to maximize placebo effects (21, 24, 51). However, when compared with female participants, males exhibited a different pattern: in High expectancy group, they reported a greater reduction of pain intensity/unpleasantness due to placebo treatment. These findings suggested that a placebo response to an expectancy manipulation can vary tremendously by gender. However, we failed to observe the main effect of gender in placebo responses, which is inconsistent with a few previous studies (33, 34, 52). Admittedly, the issue of gender discrepancy in placebo responses is still controversial, and further investigation on this issue is highly needed. Please note that gender-specific placebo effects would have tremendous implications for medical research and clinical conditions, such as pain and neurological disorders, in which placebo responses are commonly considered relevant (53, 54).

Secondly, we provided evidence showing that placebo effects, defined as the reduction of pain unpleasantness, were influenced by the interaction between expectancy level and other personal characteristics, such as dispositional optimism and anxiety state. Previous evidence proved that individuals with high scores of dispositional optimism or low scores of anxiety state were more likely to respond to placebo treatment (36, 37). This is in line with our results demonstrating that optimists (those with high LOT-R scores) or participants with low STAI-S scores showed greater placebo responses after treatments. Obviously, being more optimistic or less anxious has a positive influence on the experience to a placebo treatment, as these individuals have a tendency to hold positive expectation (55). In particular, the present results might help explain the consistent correlation between dispositional optimism and positive medical outcomes (56–58). It is suggested that the different respondence between optimists and pessimists (those with low LOT-R scores) to placebo-related expectations may contribute to placebo response discrepancy. Noted that such an effect of dispositional optimism on placebo effects was confirmed in our study, but only observed in Low expectancy group, suggesting that a realistically reasonable, but not an overly-positive expectancy could optimize the influence of dispositional optimism on the placebo response. In other words, since optimists cannot be frequently driven by negative expectancy as forcefully as pessimists can, they might experience fewer negative events. Further, may it be the optimists, not the pessimists, who could be most likely to respond to a placebo-related expectancy for positive outcomes. The above speculation is consistent with a study on persuasion, in which optimists were more likely than pessimists to be persuaded by positively structural arguments (59). Therefore, an individual with high dispositional optimism might not only be less susceptible to negative expectancy, but also be more possible than those with lower dispositional optimism to benefit from positive expectancy, particularly at realistically optimized expectancy level. This is also prompted that patients with high dispositional optimism should be informed more frequently about a certain treatment with realistically positive expectancy to strengthen their responses in medical care. Future studies are needed to explore this issue under clinical conditions.

Importantly, in line with previous studies demonstrating that people's belief can be influenced by personal characteristics, such as optimism, neuroticism, and extraversion (25, 26, 60), our observation provides further evidence suggesting that the placebo effect can be jointly affected by the expectancy level and personal characteristics, which is fitted well with the Response Expectancy Theory (24, 51). Notably, there are tremendous differences in personal characteristics between healthy population and patients. For example, depressive, persistent social phobic, neurotic, fearful, and obsessive-compulsive personality characteristics are very common in pain sufferers compared to healthy population, whereas patients undergoing injectable aesthetic treatments scored significantly higher on extraversion, agreeableness, openness to experience, and neuroticism (61–63). Thus, the next important step is to replicate the main findings of the present study in clinical conditions. To note, a growing body of neurobiological researches on placebo effects indicated the influence of cognitive progressing on the modulation of pain perception (64, 65), which implied that an integrated model combining cognitive factors with psychological factors is warranted to comprehensively explore the placebo mechanisms.

## Limitations

There are two limitations in the present study. First, although we assigned both male and female experimenters randomly to the participants, we did not control the potential influence of experimenters' gender well-enough, which still could have increased error variance. Selecting either a male or a female experimenter might be more suitable for further investigations. Second, we examined the effect of the interaction between expectancy level and personal characteristics on placebo effects within a non-clinical population, and it calls for clinical studies to replicate the main findings of the present study.

## Conclusion

Considering that placebo effects have been recognized as effective psychobiological events attributing to the improvement of the overall therapeutic outcomes, we believe that our findings not only advance our understanding of the psychological underpinnings of placebo effects, but also suggest a constructive way (regulating the expectation level individually based on the assessment of personal characteristics) to maximize placebo effects in various clinical applications.

## Ethics Statement

This experiment was approved by the Ethics Committee of Southwest University, China, and registered with ChiCTR1800014737 through Chinese Clinical Trial Register Centre. All procedure was carried out in accordance with the relevant approved lines.

## Author Contributions

HW, LW, XJL, and LH conceived and designed the experiments. HW and HZ performed the experiments. LZ, XJL, and LH analyzed the data. LZ, HW, XYL, CB, LW, XJL, and LH wrote the paper. All authors approved the final manuscript and agreed to be accountable for all aspects of the work.

### Conflict of Interest Statement

The authors declare that the research was conducted in the absence of any commercial or financial relationships that could be construed as a potential conflict of interest.
